# Case Report: Addition of PD-1 Antibody Camrelizumab Overcame Resistance to Trastuzumab Plus Chemotherapy in a HER2-Positive, Metastatic Gallbladder Cancer Patient

**DOI:** 10.3389/fimmu.2021.784861

**Published:** 2022-01-06

**Authors:** Li Wang, Xiaomo Li, Yurong Cheng, Jing Yang, Si Liu, Tonghui Ma, Li Luo, Yanping Hu, Yi Cai, Dong Yan

**Affiliations:** ^1^ Department of Oncology, Beijing Luhe Hospital Affiliated to Capital Medical University, Beijing, China; ^2^ Department of Translational Medicine, Genetron Health (Beijing) Technology, Co. Ltd, Beijing, China; ^3^ Department of Pathology, Beijing Luhe Hospital Affiliated to Capital Medical University, Beijing, China; ^4^ Independent Researcher, Ellicott City, MD, United States

**Keywords:** gallbladder cancer, *HER2* amplification, trastuzumab resistance, combination immunotherapy, camrelizumab, irAE, case report

## Abstract

*HER2* amplification/overexpression is a common driver in a variety of cancers including gallbladder cancer (GBC). For patients with metastatic GBC, chemotherapy remains the standard of care with limited efficacy. The combination of HER2 antibody trastuzumab plus chemotherapy is the frontline treatment option for patients with HER2-positive breast cancer and gastric cancer. Recently, this regime also showed antitumor activity in HER2-positive GBC. However, resistance to this regime represents a clinical challenge. Camrelizumab is a novel PD-1 antibody approved for Hodgkin lymphoma and hepatocellular carcinoma in China. In this study, we presented a HER2-positive metastatic GBC patient who was refractory to trastuzumab plus chemotherapy but experienced significant clinical benefit after the addition of camrelizumab. Our case highlights the potential of immunotherapy in combination with HER2-targeted therapy in HER2-positive GBC. We also demonstrated that two immune-related adverse events (irAEs) associated with camrelizumab can be managed with an anti-VEGF agent apatinib. This case not only highlights the importance of irAE management in patients treated with camrelizumab, but also demonstrates the potential of PD-1 antibody plus trastuzumab in HER2-positive GBC patients who have developed resistance to chemotherapy and trastuzumab-based targeted therapy.

## Introduction

Biliary tract cancers (BTCs) are low-incidence epithelial malignancies in the biliary tree, including gallbladder cancer (GBC) and cholangiocarcinoma (CCA). From 1990 to 2017, the global incidence and mortality of BTCs increased by 76% and 65%, respectively (1). Recently, FGFR inhibitors (pemigatinib and infigratinib) and IDH1 inhibitor ivosidenib have significantly improved the outcomes of CCA patients harboring *FGFR2* or *IDH1* alterations, respectively (2). The FDA approvals of these agents and the endorsement from the latest NCCN guideline demonstrated that the treatment of CCA finally enters the era of precision therapy (3). In contrast, chemotherapy is the only systemic treatment available for advanced or metastatic GBC patients, and its clinical benefit is limited, with a median overall survival (OS) of less than one year (2). Given the dismal prognosis of GBC patients, a biomarker-guided personalized treatment strategy should be explored in this BTC subtype without targeted therapy or immunotherapy options (3, 4).

Genomic profiling studies revealed that the amplification or overexpression of *ERBB2/HER2* is a major targetable mutation in GBC (5–7). HER2 is a member of the ERBB family of receptor tyrosine kinases and an established therapeutic target in breast, gastric and gastroesophageal junction (GEJ) cancers (8). A variety of HER2-directed agents including monoclonal antibodies, tyrosine kinase inhibitors, and antibody-drug conjugates (ADCs), have significantly improved the outcomes of patients with HER2-positive breast cancer and become the front-line treatment options for this disease (8).

Cancer immunotherapy agents such as immune checkpoint inhibitors have caused a paradigm shift in the landscape of cancer treatment. In addition to monotherapy, immune checkpoint inhibitors combined with HER2-directed therapies, provide a promising strategy to combat trastuzumab resistance in various HER2-positive cancers. For instance, in the phase 1b/2 PANACEA trial, the combination of PD-1 antibody pembrolizumab and trastuzumab showed activity and durable clinical benefit in patients with trastuzumab-resistant, HER2-positive breast cancer (9). Similarly, pembrolizumab plus trastuzumab and chemotherapy showed superior efficacy in HER2-positive gastric/GEJ cancers, which resulted in the accelerated approval of this triplet regime as the first-line treatment by the FDA (10). In this study, we encountered a HER2-positive, metastatic GBC patient refractory to chemotherapy and HER2-targeted therapy plus chemotherapy. Surprisingly, he responded to a series of combination therapies containing trastuzumab plus a novel PD-1 antibody camrelizumab. Our results suggest that the combination of PD-1 antibody plus trastuzumab with or without chemotherapy could be feasible treatment options for trastuzumab-resistant, HER2-positive, advanced GBC.

## Case Presentation

**Figure 1:**
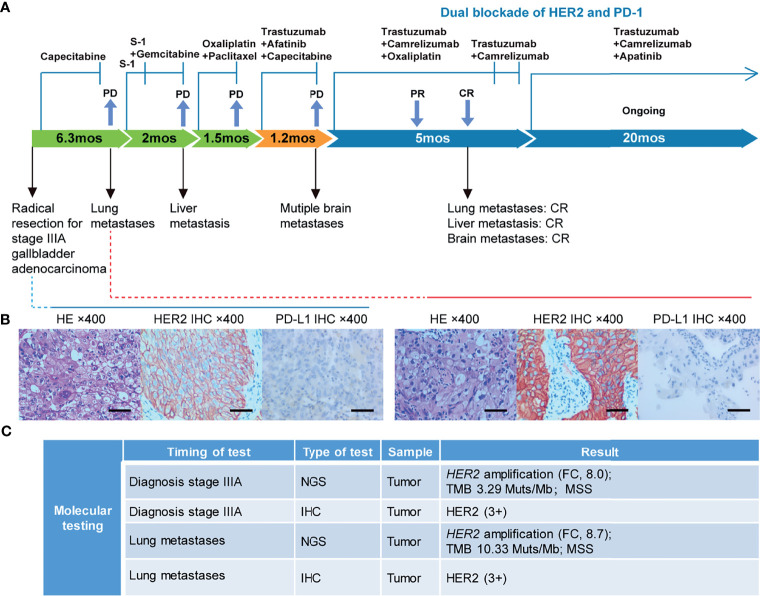
Case summary. **(A)** Summary of disease course, treatment procedure, and key molecular findings. PR, partial response; CR, complete response; PD, progressive disease; mo, months. **(B)** H&E, HER2, and PD-L1 staining of the primary tumor and lung metastasis. Scale bars: 25 µm. H&E: hematoxylin and eosin. **(C)** Detailed molecular alterations of the primary tumor and lung metastasis. FC, fold change; MSS, microsatellite stable; TMB, tumor mutational burden.

In September 2018, a 67-year-old Chinese man was admitted to our hospital due to a gallbladder mass revealed by ultrasonography during routine physical examination. His baseline Karnofsky Performance Status (KPS) was 100. Radical resection was performed on October 9^th^, 2018, and postoperative pathology revealed a stage IIIA (T3N0M0) moderately-differentiated GBC. A summary of his treatment history is illustrated in **Figure 1A**. Immunohistochemical (IHC) staining was positive for HER2 (3+) and negative for PD-L1 (**Figure 1B**). The patient received five cycles of capecitabine. A follow-up chest CT scan in April 2019 revealed disease progression in the lung and his KPS score dropped to 80. Biopsy of a pulmonary lesion confirmed staged IV (T3N0M1) GBC. IHC staining was positive for HER2 (3+), CK8/18, CK19, AE1/AE3 and negative for PD-L1, TTF-1, Napsin-A, p40, and p63. Treatment was changed to S-1 monotherapy for one cycle before the addition of gemcitabine. After three cycles, a new liver lesion and increased bilirubin level were noted. Treatment was switched to oxaliplatin and paclitaxel, but left off after one cycle due to disease progression, as well as multiple adverse events including limbs numbness, grade 2 peripheral neurotoxicity, grade 3 diarrhea, increased γ-glutamyltranspeptidase level, and severe abdominal pain (numeric rating scale of 8-9).

**Figure 2:**
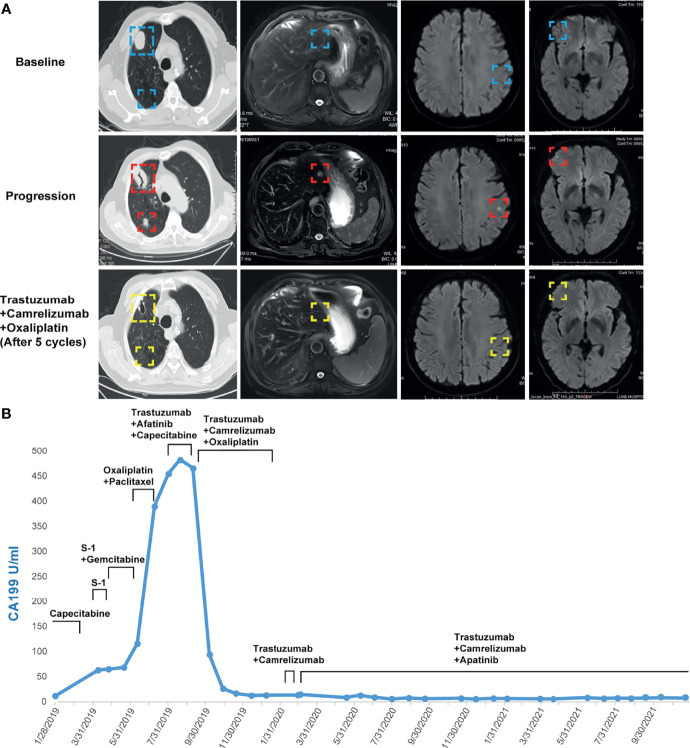
**(A)** Computed tomography and MRI images showing the patient’s baseline disease, progression on S-1 plus gemcitabine in the liver metastatic lesion, progression on trastuzumab plus afatinib and capecitabine in the brain and lung metastatic lesions, and response to camrelizumab plus trastuzumab and oxaliplatin in the liver, brain, and lung metastatic lesions. **(B)** Dynamics of cancer antigen 199 (CA199) (U/ml) levels during the entire disease course.

The failure of chemotherapy led us to explore the possibility of targeted therapy. A multi-gene next-generation sequencing (NGS) testing (Onco Panscan™, Genetron Health) was performed on the primary lesion and a pulmonary nodule to identify potential therapeutic targets (**Figure 1C** and **Supplemental Table 1**). Genomic profiling of the pulmonary lesion showed the presence of *TP53* S241Y, *ARID2* R273*, *EGFR* E872K mutations, *HER2* amplification (fold change, 8.7), and a high tumor mutational burden (TMB) of 10.33 mutations per megabase (mut/Mb). The *EGFR* E872K mutation, originally found in a bile duct carcinoma patient, was associated with activation of EGFR signaling, a common mechanism for acquired trastuzumab resistance in HER2-positive esophagogastric (EG) cancer (11, 12). To target *HER2* amplification and putative activation of EGFR signaling mediated by the *EGFR* E872K mutation, this patient was treated with a triplet regime consisting of trastuzumab (6mg/kg, Q3W), HER2/EGFR inhibitor afatinib (30mg, QD), and capecitabine (2500mg/m^2^ on days 1-14, Q3W) in August 2019. In the first dose of trastuzumab infusion, the patient experienced chills and fever, which were resolved by antipyretic treatment. In September 2019, this combination therapy was discontinued when new brain metastases and pulmonary progression of disease were noted (**Figure 2A**).

**Figure 3:**
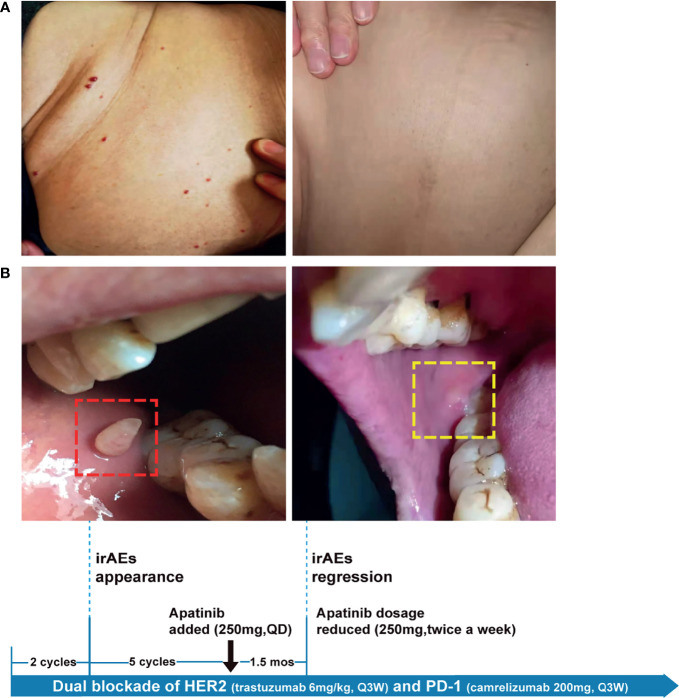
The management of camrelizumab-related irAEs with anti-VEGF agent apatinib. Representative images showing irAEs of **(A)** RCCEP, **(B)** mucosal membrane and their resolution after apatinib treatment. irAE, immune-related adverse event; RCCEP, reactive cutaneous capillary endothelial proliferation.

Because HER2-targeting antibody-drug conjugates (ADCs) such as ado-trastuzumab emtansine (T-DM1) and fam-trastuzumab deruxtecan (T-DXd) were not approved in China at that time, we set out to explore the possibility of immunotherapy given the TMB-H status of the pulmonary metastases. In the basket study of the KEYNOTE-158 trial, pembrolizumab showed durable antitumor activity in a subset of patients with advanced biliary adenocarcinoma irrespective of PD-L1 status (13). In a single-center phase 2 trial, the combination of pembrolizumab plus trastuzumab and chemotherapy achieved a disease control rate of 100% and an objective response rate (ORR) of 83% in HER2-positive metastatic esophagogastric adenocarcinoma irrespective of PD-L1 status (14). Based on these results and the affordability of a domestic PD-1 antibody camrelizumab, a triplet regime of camrelizumab (200mg, Q3W), trastuzumab (6mg/kg, Q3W), and oxaliplatin (130mg/m^2^, Q3W) were administered for six cycles. In the first cycle, radical cryoablation was performed to treat the lung metastases. After three cycles, regression of all metastases involving multiple organs was noted. The lesions of the lung, liver, and brain completely regressed after five cycles (**Figure 2A**). The patient’s CA199, which continued to increase during chemotherapy and HER2-directed therapy, quickly fell into the normal range after the addition of camrelizumab, indicative of response to the combination regime (**Figure 2B**). Oxaliplatin was discontinued after six cycles due to grade 2 thrombocytopenia, which was subsequently resolved with recombinant human interleukin-11 (rhlL-11) treatment. The patient continued on maintenance camrelizumab plus trastuzumab, with a KPS score of 90. After cycle 2 of camrelizumab, the patient developed grade 1 reactive cutaneous capillary endothelial proliferation (RCCEP), a novel immune-related adverse event (irAE) observed in the majority of patients treated with camrelizumab (15, 16). The symptoms of RCCEP reached grade 2 in cycles 3-4 when the patient developed one cutaneous nodule with a diameter larger than 10mm and bleeding. Apatinib, a small-molecule VEGFR2 inhibitor, has been approved to treat gastric cancer in China (17). The addition of low-dose apatinib to camrelizumab significantly reduced the frequency of RCCEP in clinical studies (18–20). Based on these results, apatinib (250mg, QD) was added to the treatment regime. The patient’s RCCEP became grade 1 on week 3, and completely regressed on week 6 (**Figure 3A**). Interestingly, the addition of apatinib also led to the complete resolution of an endothelial neovessel-based nodule in the right buccal mucosa, a putative camrelizumab-related irAE (**Figure 3B**). Apatinib was then decreased to 250mg, twice a week with no recurrence of RCCEP. The patient remained in remission until the last follow-up in November 2021 with a KPS score of 90.

## Discussion

Biliary tract cancers (BTCs), including cholangiocarcinoma (CCA) and gallbladder cancer (GBC), are a group of gastrointestinal cancers with low incidence and poor prognosis (21). BTCs are generally refractory to chemotherapy and the 5-year survival rate of BTC patients ranges from 5% to 15% (22). HER2 overexpression is detected in 13%-31% of GBC cases and is a promising candidate for targeted therapy clinical trials (23–25). HER2 is an established therapeutic target in HER2-positive breast, gastric, and gastroesophageal junction (GEJ) cancers (8, 26). Multiple HER2-directed agents have been approved to treat HER2-positive breast cancer, including trastuzumab, pertuzumab, margetuximab, trastuzumab emtansine (T-DM1), trastuzumab deruxtecan (T-DXd/DS-8201), neratinib, lapatinib, and tucatinib (27). However, the options of HER2-targeted therapies for HER2-positive gastrointestinal cancers are very limited. For instance, only trastuzumab and trastuzumab deruxtecan are approved for HER2-positive gastric/GEJ cancer (28). Unlike HER2-positive breast cancer and gastric/GEJ cancer, there is no HER2-directed therapy approved for HER2-positive BTC. Multiple HER2-directed agents, including receptor tyrosine kinase inhibitors, monoclonal antibodies, and antibody-drug conjugates, have been or are currently being tested in ongoing trials for patients with BTC. Lapatinib, a HER2 receptor tyrosine kinase inhibitor (TKI), failed to show activity in two phase 2 trials for unselected patients with BTC (29, 30). In the phase 2 SUMMIT basket trial, neratinib achieved an ORR of 12% and a median PFS of 2.8 months in 25 patients with *HER2*-mutated BTC (31). The combination of trastuzumab and pertuzumab resulted in an ORR of 23% in a multicenter, open-label, phase 2a trial for HER2-positive, metastatic BTC (32). In a phase 2 basket trial (NCT02675829), trastuzumab emtansine (T-DM1) resulted in an ORR of 16.7% in 6 patients with *HER2*-amplified BTC (7). In a phase 1 trial for pretreated, HER2-expressing (IHC > 1+), non-breast/non-gastric solid tumors, trastuzumab deruxtecan achieved partial response in two BTC patients (33).

Cancer immunotherapy has led to a paradigm shift in cancer treatment. Recent clinical studies reported a synergistic effect of the combination of immune checkpoint inhibitors with HER2-directed agents in HER2-positive gastric/GEJ cancers (34, 35). Interim analysis of the ongoing KEYNOTE-811 trial showed that the triplet regime of pembrolizumab, trastuzumab, and chemotherapy resulted in a substantial, statistically significant increase in ORR versus the duplet regime of trastuzumab and chemotherapy as first-line therapy for HER2-positive metastatic gastric/GEJ cancer (10). Based on these results, FDA granted accelerated approval of pembrolizumab in combination with trastuzumab and chemotherapy as the first-line treatment for patients with locally advanced unresectable or metastatic HER2 positive gastric/GEJ cancer.

Currently, PD-L1 is the most widely used biomarker to guide the selection of patients to receive PD-1/PD-L1 immune checkpoint blockade therapy (36). One intriguing observation of this case study is that the clinical response to PD-1/HER2 dual blockade did not correlate with the PD-L1-negative status of the primary tumor and lung metastases. Interestingly, in some PD-1/HER2 dual blockade trials of HER2-positive gastrointestinal cancers, the clinical benefit did not correlate with PD-L1 status either. For instance, in the CP-MGAH22–05 phase 1b/2 trial for HER2-positive gastroesophageal carcinoma patients, pembrolizumab plus HER2 antibody margetuximab resulted in disease control rate of 72% and 56% in HER2 IHC-positive/PD-L1-positive and HER2 IHC-positive/PD-L1-negative patients, respectively (34). Similarly, in the phase 2 trial testing the efficacy of pembrolizumab plus trastuzumab in patients with HER-2 positive esophagogastric cancer, the median PFS in the PD-L1-negative group was numerically longer than that of the PD-L1-positive group (14.6 versus 10.3 months, respectively; p = 0.56) (35). Together, these results indicated that HER2-positive GBC patients with PD-L1-negative status should be enrolled in future clinical trials of dual PD-1/HER2 blockade therapy as well.

Tumor mutational burden (TMB) is a useful but imperfect predictive biomarker for cancer immunotherapy. Based on results of the KEYNOTE-158 trial, FDA recently approved pembrolizumab for patients who have solid tumors with a TMB greater than 10 mut/Mb (37). This threshold was determined by the FoundationOne CDx (F1CDx) assay, which targets a genomic region of ~0.8 Mb covering 324 cancer-related genes (38). The gold standard to measure TMB is whole exome sequencing (WES), which is impractical to use in the clinic right now. For accurate TMB assessment, multi-gene panels covering at least 1 Mb are generally recommended (38, 39). The phase 2 KEYNOTE-158 trial covered 10 different rare cancers including biliary adenocarcinoma (37). Whether the results of these rare cancers can be extended to common cancers is a controversial issue (40, 41). It is recommended that the application of TMB as an immune biomarker should be cancer-type specific and in combination with other immune biomarkers (38–40). Further investigations are required to fully explore the potential of TMB as an immune biomarker for GBC treatment.

The timely recognition and mitigation of serious immune-related adverse events (irAEs) are essential for the optimal management of cancer patients treated with ICIs. A novel ICI-related irAE, reactive cutaneous capillary endothelial proliferation (RCCEP), represents the most common irAE associated with camrelizumab (42–44). To address this issue, the Chinese Society of Clinical Oncology (CSCO) recently developed an RCCEP management guideline, which recommended special interventions for grade 2 RCCEP with large nodules or bleeding (45). Although the mechanism of RCCEP is still unknown, it was observed that RCCEP correlates with increased expression of VEGF-A and activation of VEGFR-2 receptor, two key players of VEGF signaling (45). Interestingly, a retrospective meta-analysis revealed that the median resolution time of RCCEP was 6.5 months in patients treated with camrelizumab alone but 2.2 months if camrelizumab was combined with anti-VEGF therapy (45). Consistently, several clinical studies showed that the addition of VEGFR-2 inhibitor apatinib significantly reduced the frequency of RCCEP or alleviated symptoms of RCCEP in patients treated with camrelizumab (46–49). Together, these results indicated that anti-VEGF therapy could be a promising strategy for the management of camrelizumab-related RCCEP.

In summary, we applied a triplet combination of camrelizumab, trastuzumab, and chemotherapy to overcome the resistance to chemotherapy and HER2-targeted therapy in a HER2-positive GBC patient. The response was maintained after the withdraw of chemotherapy. Our case demonstrated that the combination of PD-1 antibody plus trastuzumab with or without chemotherapy can produce robust and durable responses in metastatic HER2-positive GBC resistant to trastuzumab and chemotherapy. Some of the camrelizumab-related irAEs may be managed by the anti-VEGF agent apatinib. Future investigation of PD-1 antibody plus trastuzumab with or without chemotherapy as the frontline treatments for HER2-positive GBC is warranted.

## Data Availability Statement

The original contributions presented in the study are included in the article/**Supplementary Material**. Further inquiries can be directed to the corresponding authors.

## Ethics Statement

Written informed consent was obtained from the individual(s) for the publication of any potentially identifiable images or data included in this article.

## Author Contributions

DY, conceptualization, methodology, supervision, writing-reviewing and editing. YC, conceptualization, investigation, and writing-original draft preparation. LW and XL, methodology, visualization, investigation, and writing-original draft preparation. YRC, JY, and SL, investigation, data curation, and validation. TM, validation, writing-reviewing and editing. LL and YH, methodology and validation. All authors contributed to the article and approved the submitted version.

## Conflict of Interest

XL, SL, and TM are employees of Genetron Health (Beijing) Technology, Co. Ltd.

The remaining authors declare that the research was conducted in the absence of any commercial or financial relationships that could be construed as a potential conflict of interest.

## Publisher’s Note

All claims expressed in this article are solely those of the authors and do not necessarily represent those of their affiliated organizations, or those of the publisher, the editors and the reviewers. Any product that may be evaluated in this article, or claim that may be made by its manufacturer, is not guaranteed or endorsed by the publisher.
