# Supplementary material for: Case Report: Addition of PD-1 Antibody Camrelizumab Overcame Resistance to Trastuzumab Plus Chemotherapy in a HER2-Positive, Metastatic Gallbladder Cancer Patient
Source: Front Immunol. 2022 Jan 6;12:784861. doi: 10.3389/fimmu.2021.784861 (PMC8770537; doi:10.3389/fimmu.2021.784861)
Supplement: Supplementary file 1 [file Table_1.docx]

Supplemental Table 1. Genetic alterations in primary tumor and lung metastasis

| Gene | Nucleotide change | Amino acid change | Mutation effect | Primary tumor  VAF (%) | Lung metastasis  VAF (%) |
| --- | --- | --- | --- | --- | --- |
| *EGFR* | c.2614G>A | p.E872K | Nonsynonymous | 7.4 | 13.5 |
| *FH* | c.739-1G>A | - | Splicing | 5.2 | 10.5 |
| *HIST1H3A* | c.104G>A | p.G35D | Nonsynonymous | 6.4 | 14.6 |
| *HIST1H3B* | c.220G>C | p.E74Q | Nonsynonymous | 5.2 | 12.7 |
| *TP53* | c.722C>A | p.S241Y | Nonsynonymous | 5.4 | 13.1 |
| *TRRAP* | c.9487G>A | p.E3163K | Nonsynonymous | 5.3 | 13.0 |
| *TUBG1* | c.443G>C | p.G148A | Nonsynonymous | 5.8 | 8.9 |
| *USHBP1* | c.358C>G | p.Q120E | Nonsynonymous | 5.9 | 17.3 |
| *ACVR2A* | c.214G>A | p.E72K | Nonsynonymous | - | 5.1 |
| *ARID2* | c.820C>T | p.R274* | Stopgain | - | 9.7 |
| *ARID2* | c.4793C>T | p.S1598L | Nonsynonymous | - | 11.5 |
| *CFHR1* | c.194C>T | p.S65L | Nonsynonymous | - | 8.0 |
| *GATA1* | c.464C>T | p.S155L | Nonsynonymous | - | 11.6 |
| *MDM2* | c.1096G>A | p.E366K | Nonsynonymous | - | 14.1 |
| *PAK1* | c.256C>T | p.H86Y | Nonsynonymous | - | 11.0 |
| *PTPRD* | c.3232G>C | p.E1078Q | Nonsynonymous | - | 6.8 |
| *SDC4* | c.166G>C | p.D56H | Nonsynonymous | - | 5.2 |
| *SPTA1* | c.795C>A | p.N265K | Nonsynonymous | - | 10.2 |
| *TRPS1* | c.2920G>A | p.E974K | Nonsynonymous | - | 8.9 |
| *XPC* | c.2745G>C | p.K915N | Nonsynonymous | - | 8.6 |
| MSI |  |  |  | MSS | MSS |
| TMB |  |  |  | 3.29mutations/MB | 10.33mutations/MB |

VAF: variant allele frequency; MSI: microsatellite instability; MSS: microsatellite stable; TMB: tumor mutational burden.
